# Acute kidney injury and hepatitis associated with energy drink consumption: a case report

**DOI:** 10.1186/s13256-019-2340-0

**Published:** 2020-01-29

**Authors:** Raed Al Yacoub, Debra Luczkiewicz, Christopher Kerr

**Affiliations:** 1 Division of Hospital Medicine, Department of Internal Medicine, University of Florida, 1230 SW 11th Ave, C405, Gainesville, FL 32601 USA; 2 Division of Geriatric and Palliative care, Department of Internal Medicine, University at Buffalo, Buffalo, NY USA; 3 The Center for Hospice & Palliative Care, Cheektowaga, NY USA

**Keywords:** Energy drinks (EDs), Acute kidney injury (AKI), Acute hepatitis (AH)

## Abstract

**Introduction:**

In the USA, energy drinks are commonly consumed among adults. The side effects of these drinks are not well studied but consumers have reported multiple adverse events to the US Food and Drug Administration including acute kidney injury and acute hepatitis.

**Case presentation:**

A 62-year-old white woman presented with progressive weakness, fatigue, confusion, and delirium secondary to acute kidney injury and acute hepatitis associated with excessive energy drink use. Clinical improvement occurred with supportive care and discontinuation of energy drinks, with resolution of acute kidney injury and progressive improvement of liver function. The defined mechanism of injury is unknown but thought due to energy drink ingredients.

**Conclusion:**

Multiple cases of energy drink-induced acute kidney injury or acute hepatitis are reported in the literature but this case is the first to report them simultaneously. Ingredients and presumed doses to cause these events are outlined in this case report.

## Introduction

The consumption of energy drinks (EDs) increased from 2003 to 2016 in all age groups, including middle-aged (45 to 59-year old) adults, whose consumption increased from 0.0% to 1.2% [1]. Many adverse event reports received by the US Food and Drug Administration from consumers through 2012 include psychiatric symptoms, arrhythmias, cardiac arrest, myocardial infarction, convulsions, and renal and liver impairment [2]. The mechanisms of injury are not well studied. The ingredients of EDs vary but most of them contain caffeine, L-carnitine, taurine, B vitamins, glucuronolactone, antioxidants, trace minerals, guarana, sucrose, *Ginkgo biloba*, and/or ginseng, some of which act as stimulants [3].

Previous case reports revealed acute kidney injury (AKI) induced by excessive ED consumption thought to be due to taurine, [4, 5] while others reported acute hepatitis (AH) attributed to niacin [6–8]. The doses that caused the injuries varied and are probably due to interactions with other ingredients.

## Case presentation

A 62-year-old white woman who had been enrolled in hospice care for 4 months since discontinuing treatment for small cell carcinoma of the left lung presented to the hospice in-patient unit with several days’ history of progressive confusion, fatigue, poor sleep, decreased intake, nausea, and vomiting. On initial assessment her condition was thought to most likely stem from progression of her cancer. She was treated symptomatically for nausea and delirium, but continued to decline, developing diaphoresis, decreased level of consciousness, increased weakness, and lethargy. Further history revealed that over several weeks prior to admission her appetite had declined with minimal intake except for five to six cans of a 16 fluid ounce sugar-free ED daily.

On day 3, laboratory tests revealed significant hepatic and renal dysfunction. Baseline kidney and liver tests had been within normal range 2 months previously, except for mildly elevated alkaline phosphatase (ALP) (Table 1). A chest X-ray showed no acute cardiopulmonary disease. She received hydration with normal saline, empiric treatment of infection with ceftriaxone because of elevated white blood cell (WBC) count, and her home medications were adjusted for liver and kidney functions. Repeat laboratory tests on day 6 showed slightly improved liver but worsening renal function (Table 1). A urine culture was negative, and WBC normalized. Ultrasound revealed normal liver echogenicity, normal gallbladder with wall thickness 2 mm, mild extrahepatic and intrahepatic duct dilatation (seen on previous imaging), and normal kidneys.

**Table 1:** Laboratory results

Blood test	2 months prior	Day 3	Day 6	Day 10	2 months after	Normal range
WBC × 10^9^/L	11	15.3	10.6	14.4	10	4–11
HgB (g/dL)	14.2	15.6	13.3	13	15.2	12.5–15.5
Platelet × 10^9^/L	237	133	78 (mild clumping)	133	237	150–450
sCr. (mg/dL)	0.77	2.83	4	0.72	0.69	0.6–1.2
BUN (mg/dL)	13	18	85	23	12	8–27
GFR (ml/minute/1.72 m^2^)	> 60	17	11	89	> 90	>90
Total bilirubin (mg/dL)	0.3	0.5	0.5	0.8	0.3	0.3–1
AST (U/L)	39	4333	1129	70	30	13–39
ALT (U/L)	30	2866	2928	812	41	7–52
Alkaline phosphatase (U/L)	121	111	113	88	110	34–104
Ammonia (μmol/L)			149	89		16–53

*ALT* alanine aminotransferase, *AST* aspartate aminotransferase, *BUN* blood urea nitrogen, *GFR* glomerular filtration rate, *HgB* hemoglobin, *sCr*. serum creatinine, *WBC* white blood cell

The family confirmed our patient’s wishes to avoid transfer to the hospital or aggressive interventions such as dialysis or further intravenously administered antibiotics and was accepting of the possibility of limited prognosis. Supportive care was provided with hydration, parenteral medications, and symptom management. On days 8–9, she became more alert and began to take food, fluids, and medications reliably by mouth. Repeat laboratory tests on day 10 showed significant improvement consistent with her clinical condition with normal renal function and greatly improved liver enzymes. She returned to her baseline mental and functional status and was discharged home on day 14 with instructions to avoid further consumption of any ED products.

## Discussion

As patients enrolled in hospice care begin to decline they often seek ways to improve or maintain hydration. EDs differ from “sport drinks” which provide hydration and replete electrolytes [3]. EDs contain high levels of carbohydrates, which affect fluid absorption and cause gastrointestinal distress, and they contain caffeine, which causes diuresis leading to increased urinary output and natriuresis instead of hydration [3]. Unfortunately, there are limited studies about the long-term effects of ED ingredients in humans [3]. Table 2 lists the ingredients in the ED consumed by our patient, who was drinking five to six cans (10–12 servings) per day.

**Table 2:** Energy drink ingredients

	Per serving (8 oz. or 240 ml)
Taurine	1000 mg
Guarana seed extract	100 mg
Caffeine	80 mg
Glucuronolactone	50 mg
L-carnitine	25 mg
B8 (inositol)	25 mg
B3 (niacin)	20 mg
B6	2 mg
B12 (cyanocobalamin)	2 microgram
B2 (riboflavin)	3.4 mg
B5 (pantothenic acid)	10 mg

Our patient appeared to develop AKI and AH simultaneously. She denied use of herbal supplements or alcohol; she said she had previously tested negative for viral hepatitis, had no new medications or recent imaging with contrast, and was not on nephrotoxic medications. Her strongest risk factor was her daily consumption of large amounts of EDs. Based on a review of the literature (Table 3), the main contributor to AKI was most probably taurine, and, for AH, niacin, although other ingredients or combinations of ingredients may also have played a role.

**Table 3:** Summary of literature on the adverse effects of energy drink consumption

Reference	Case summary	Conclusion
1- Schöffl *et al*., 2011 [4]	According to Schöffl *et al*., Lehtihet M *et al*. in 2006 reported the case of a 31-year-old football referee who consumed EDs (750 ml) and developed AKI	Acute tubular necrosis and rhabdomyolysis
2-Schöffl *et al*., 2011 [4]	A 17-year-old boy consumed 3 L of EDs with 1 L of vodka (4600 mg of taurine, 780 mg caffeine, and 380 g of alcohol) with AKI	Taurine accumulation. AKI resolved in 10 days but required hemodialysis
3-Greene *et al*., 2014 [5]	A 40-year-old man consumed 100–120 oz. of EDs daily for 2–3 weeks. Presented with hypoglycemia and AKI (creatinine 5.5 mg/dL)	Taurine accumulation, creatinine returned to normal after 2 days of ED discontinuation
4-Vivekanandarajah *et al*., 2011 [6]	A 22-year-old woman consumed ten cans of an energy drink daily for 2 weeks and presented with AH. On presentation, AST, ALT, and total bilirubin were 7709 U/l, 7533 U/l, 3.5 mg/dL and on discharge day 4 they were 238 U/l, 1947 U/l, and 1.7 mg/dL, respectively	Hepatotoxicity was believed related to ED consumption, which included niacin 300 mg/day
5-Huang *et al*., 2014 [7]	A 36-year-old man consumed three 8 oz. sugar-free EDs daily for a year with a history of many years of weekend binge drinking. He presented with AH; AST 1541 U/L, ALT 2995 U/L, and bilirubin 16.1 mg/dL with continued liver impairment eventually requiring orthotopic liver transplantation	Liver biopsy consistent with herbal/drug toxicity. Niacin dose was only 120 mg per day
6-Harb *et al*., 2016 [8]	A 50-year-old man consumed four to five EDs (40 mg of niacin each) per day over 3 weeks. He presented with AH; AST 4051 U/L, AST 2073 U/L, bilirubin 19.3 mg/dL. He improved after discontinuation of EDs	Liver biopsy showed severe acute hepatitis with bridging necrosis and marked cholestasis. Daily intake of niacin was approximately 160–200 mg

*AH* acute hepatitis, *AKI* acute kidney injury, *ALT* alanine aminotransferase, *AST* aspartate aminotransferase, *EDs* energy drinks

Taurine is a sulfur-containing amino acid typically used as a nutritional supplement by athletes to increase performance. There are some data from Suliman *et al.* suggesting increased risk from taurine intake in patients with renal failure who develop neurological symptoms [9]. Suliman *et al*. also recommend avoiding EDs in these patients [9]. Our patient consumed 10–12 g/day.

Niacin (vitamin B3) can cause hepatotoxicity at doses between 1 and 5 g/day [10]. The laboratory pattern of liver injury due to drugs or toxins could be hepatocellular, cholestatic, or mixed [11]. AH has been described in several cases at lower levels of niacin consumption as a component of EDs at levels similar to our patient who consumed between 200 and 240 mg/day.

## Conclusion

To the best of our knowledge this is the first reported case of simultaneous ED induced AKI and AH. Until more research reveals safe amounts of EDs and their ingredients, excessive use should be avoided and a thorough history should include questions regarding supplements including beverages such as EDs which are perceived as safe.

## Data Availability

All the data supporting our findings is contained within the manuscript.

## Abbreviations

AH: Acute hepatitis
AKI: Acute kidney injury
EDs: Energy drinks
WBC: White blood cell
